# The application of fibular free flap with flexor hallucis longus in maxilla or mandible extensive defect: a comparison study with conventional flap

**DOI:** 10.1186/s12957-018-1450-2

**Published:** 2018-07-23

**Authors:** Youkang Ni, Ping Lu, Zhi Yang, Wenlong Wang, Wei Dai, Zhong-zheng Qi, Weiyi Duan, Zhong-fei Xu, Chang-fu Sun, Fayu Liu

**Affiliations:** Department of Oromaxillofacial-Head and Neck Surgery, School of Stomatology, China Medical University, No. 117 Nanjing North Street, Heping District, Shenyang, 110002 Liaoning People’s Republic of China

**Keywords:** Fibular free flap, Flexor hallucis longus, Maxillary and mandibular defect, Extensive defect, Reconstruction

## Abstract

**Background:**

The repair and reconstruction of maxillary and mandibular extensive defects have put huge challenges to surgeons. The fibular free flap (FFF) is one of the standard treatment choices for reconstruction. The conventional FFF has deficiencies, such as forming poor oral mucosa, limited flap tissue, and perforator vessel variation. To improve the use of FFF, we add the flexor hallucis longus (FHL) in the flap (FHL-FFF). In this paper, we described the advantage and indication of FHL-FFF and conducted a retrospective study to compare FHL-FFF and FFF without FHL.

**Methods:**

Fifty-four patients who underwent FFF were enrolled and divided into two groups: nFHL group (using FFF without FHL, 38 patients) and FHL group (using FHL-FFF, 16 patients). The perioperative clinical data of patients was collected and analyzed.

**Results:**

The flaps all survived in two groups. We mainly used FHL to fill dead space, and the donor-site morbidity was slight. In FHL group, flap harvesting time was shorter (118.63 ± 11.76 vs 125.74 ± 11.33 min, *P* = 0.042), the size of flap’s skin paddle was smaller (16.5 (0–96) vs 21.0(10–104) cm^2^, *P* = 0.027) than nFHL group. There were no significant differences (*P* > 0.05) in hospital days, hospitalization expense, rate of perioperative complications, etc. between the two groups. Compared with FFF without FHL, FHL-FFF will neither affect the use of flap nor bring more problems.

**Conclusion:**

The FHL-FFF simplifies the flap harvesting operation. The FHL can form good mucosa and make FFF rely less on skin paddle. It can be used for adding flap tissue and dealing with perforator vessel variation in reconstruction of maxillary and mandibular extensive defects.

## Background

The maxillary and mandibular extensive defects are often caused by tumor surgery, trauma, etc. The defects can cause severe functional and cosmetic deformities and have harmful effects on the patients’ quality of life. So, the patients have urgent desires to repair and reconstruct the defects.

Fibular free flap (FFF) was firstly described by Taylor in 1975 [1], and then Hidalgo [2] firstly introduced it for mandibular reconstruction in 1989. In 1993, Sadove reported simultaneous maxillary and mandibular reconstruction with one fibular free osteocutaneous flap [3]. The advantages of FFF, such as sufficient osseous tissue, reliable blood supply, precise shaping, simple flap harvesting operation, and limited donor site morbidity, make it popularly used in the clinical work [4, 5, 6]. With the development of microsurgery, it has become one of the most common methods to repair and reconstruct the maxillary and mandibular defects [6].

In our department, the conventional FFF is mainly composed of fibula and skin paddle. The skin paddle can be used as a monitoring window for the flap’s survival [7]. In practice, we find that only fibula and skin paddle are not enough to repair extensive defect. In follow-up visits, the patients always complain about the skin paddle’s discomfort in the oral cavity.

In spite of thorough presurgical planning, emergency situations that require prompt processing may arise during FFF surgeries, including perforator vessel variation and the limited flap tissue.

In 1994, Hidalgo mentioned the use of FHL in FFF for filling in the soft tissue defect in mandibular reconstruction [4]. But the indication of FHL-FFF and the comparison of FHL-FFF and FFF without FHL lack detailed description. Inspired by Hidalgo, and in order to resolve the deficiencies of the FFF and deal with the emergency during the surgery, we added flexor hallucis longus in FFF (FHL-FFF).

In this paper, we collected our FHL-FFF and FFF without FHL in the same time. We described the use of FHL in FFF and the indication of FHL-FFF. We compared FHL-FFF and FFF without FHL in patients’ clinical data and analyzed the advantages and disadvantages of the two methods. In addition, we share two cases flexibly using FHL-FFF to repair the maxillary and mandibular extensive defects.

## Methods

### Patient

**Fig. 1:**
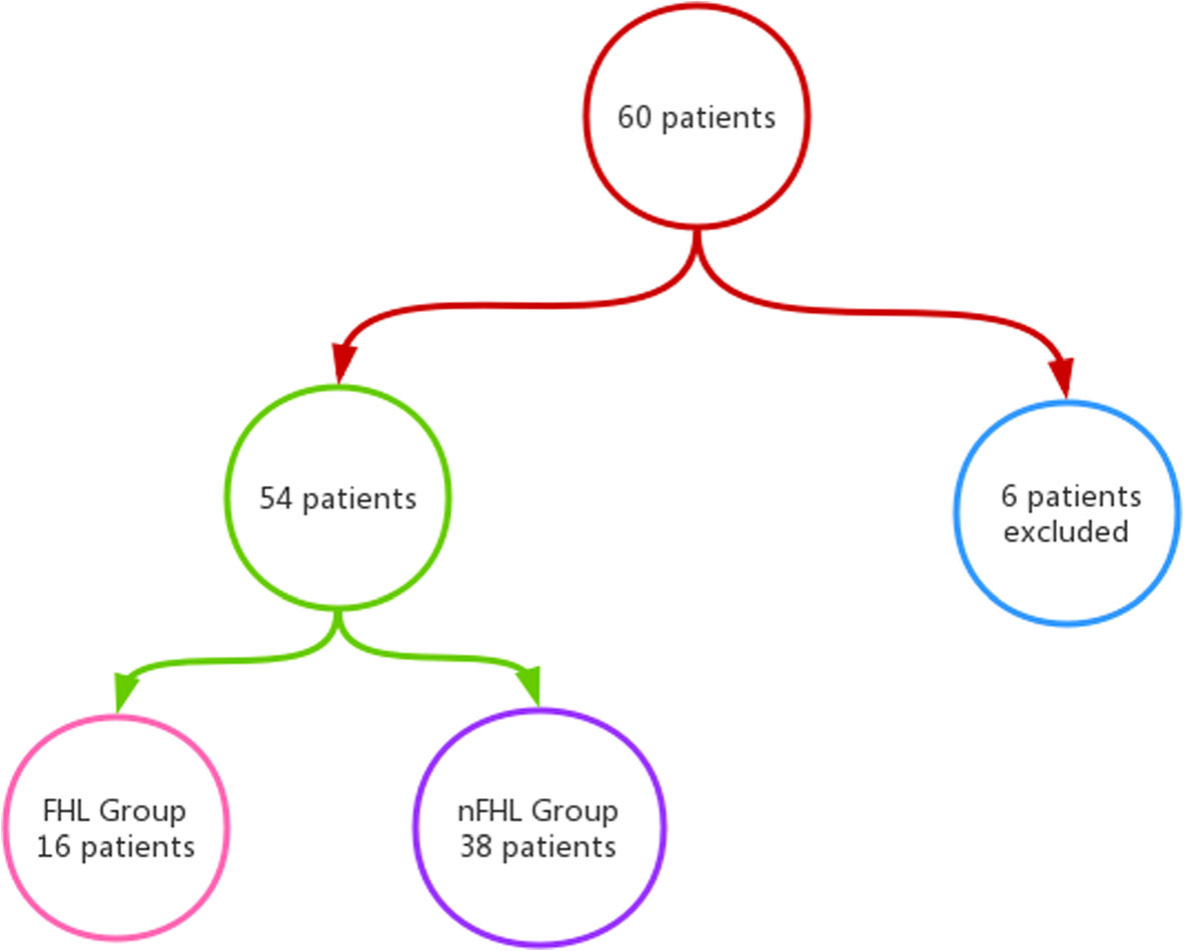
Patients’ flowchart

From Nov. 2013 to Apr. 2017, 60 patients underwent head and neck tumor resection and reconstruction with FFF at the Department of Oromaxillofacial-Head and Neck Surgery, School of Stomatology, China Medical University. The exclusion criteria are as follows: (1) the lower limb has a history of severe trauma, neuropathy, and diabetic foot; and (2) history of bone grafts more than one time. Finally, we excluded six patients and included 16 patients in FHL group, 38 patients in nFHL group (see Fig. 1).

All cases had detailed clinical records. The patient’s preoperative data include demographics, smoking and drinking history, comorbidities, etc. The operation data include time of operation, operation method and data of the flap, etc. The postoperative data include hospital stay, hospitalization expenses, etc. Patient’s informed consent was obtained, and approval was obtained from the ethics committee of the School of Stomatology, China Medical University.

Follow-up visit was conducted by telephone, Internet, and periodic review. The donor-site morbidity was observed.

### Operative technique

**Fig. 2:**
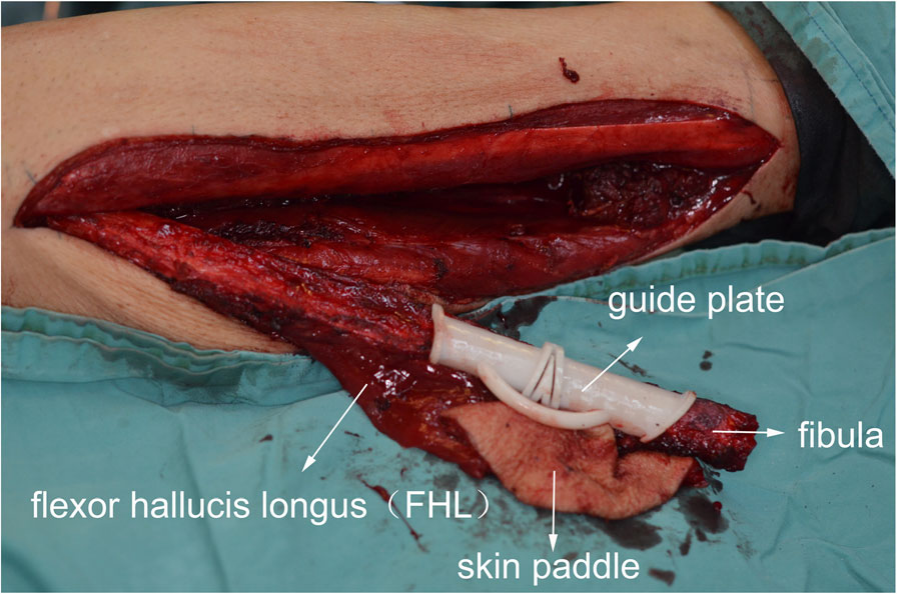
Fibula free flap with flexor hallucis longus

The primary difference between two methods is whether the FHL is harvested in the flap. The operation is under an automatic tourniquet system (ATS-III) to reduce bleeding. First, we mark the design of the flap and skin paddle on the lower extremity. Then, we take the posterolateral approach of crus to harvest the flap. After cutting and separating skin, subcutaneous and fascia tissue, we seek one or two perforator vessels and confirm the vessels are from FHL. Then, we separate peroneus longus from the fibula and interrupt intermuscular septum and bone septum. Then, the fibula is fully revealed. We could conveniently cut off the fibula. Then, we harvest the skin paddle as needed. We continue to separate tissue and seek the spatium between FHL and soleus and use finger to separate the FHL from the fibula. Finally, we only need to ligate the vessels of superior and inferior fibula, then check the fibula flap’s blooding (see Fig. 2). While in our conventional method, we need to dissect carefully the FHL from fibula and dissect peroneal artery and vein. It will prolong time of operation and increase risks of blood vessel and nerve injuries.

When we transplant the FHL-FFF to recipient site, we can use the FHL to fill the dead space after tumor resection and then use skin paddle to close defect. We can also cover the fibula with muscle and use the muscular fasciae of FHL to fill the intraoral defect.

### Statistical methods

The statistical software SPSS version 18.0 was used to analyze the data. A *P* value < 0.05 was considered statistically significant.

Patient’s age, weight, operation time, flap harvesting time, the length of harvested fibula, and hospitalization expenses obey normal distribution. To compare the differences of these items between the two groups, independent sample *T* test was used to analyze these data. Skin paddle size, total hospital days, and postoperative hospital days obey abnormal distribution. To compare the differences, Wilcoxon rank sum test was used to analyze these data. Chi-squared test was used to analyze the ratios, such as sex ratio and rate of smoking.

## Results

### Preoperative data

The FHL group had 16 patients, 15 males, and 1 female. The nFHL group had 38 patients, 18 males and 20 females. Male ratios of FHL group were significantly higher than that in nFHL group (93.75 vs 47.37%, *P* = 0.001). The mean age of FHL group was 46.44 ± 13.80 years old and nFHL group was 43.73 ± 13.66 years old. There was no significant difference in age (*P* = 0.511). The mean weight of FHL group was 66.22 ± 9.15 kg and nFHL group was 66.42 ± 13.45 kg. There was no significant difference (*P* = 0.956). The FHL group had 8 patients with smoking history and 4 patients with drinking history. The nFHL group had 9 patients with smoking history and 2 patients with drinking history. There were no significant differences in smoking and drinking rate (smoking% 50 vs 23.68%, *P* = 0.106; drinking% 25.00 vs 5.26%, *P* = 0.102). The major comorbidity was hypertension. The FHL group had 3 patients, and nFHL group had 8 patients. There was no significant difference (hypertension% 18.75 vs 21.05%, *P* = 1.000). The two groups had no history of chemoradiotherapy before the operation. In nature of tumor, the FHL group had 9 cases of benign tumor, including 8 cases of ameloblastoma and 1 case of odontogenic keratocyst; and 7 cases of malignant tumor, including 6 cases of squamous cell carcinoma and 1 case of adenoid cystic carcinoma. The nFHL group had 21 cases of benign tumor, including 16 cases of ameloblastoma, 3 cases of odontogenic myxoma, 1 case of odontogenic keratocyst, and 1 case of giant cell lesion; and 17 cases of malignant tumor, including 11 cases of squamous cell carcinoma, 1 case of clear cell carcinoma, and 5 cases of osteosarcoma. The malignant tumor rates were 56.25 and 55.26%.There was no significant difference (*P* = 0.947). In malignant tumor, the rates of carcinoma were 43.75 and 31.58%; there was no significant difference (*P* = 0.392). The rates of sarcoma were 0 and 13.16%; there was no significant difference (*P* = 0.313) (see Table 1).

**Table 1:** Preoperative data

*N*	FHL group	nFHL group	*P*
	16	38	
Sex (*n*)			
Male%	93.75% (15)	47.37% (18)	0.001*
Female%	6.25% (1)	52.63% (20)	
Age (year)	46.44 ± 13.80	43.73 ± 13.66	0.511
Weight (kg)	66.22 ± 9.15	66.42 ± 13.45	0.956
Smoking%	50.00% (8)	23.68% (9)	0.106
Drinking%	25.00% (4)	5.26% (2)	0.102
Hypertension%	18.75% (3)	21.05% (8)	1.000
Nature			
Benign%	56.25% (9)	55.26% (21)	0.947
Malignant%	43.75% (7)	44.74% (17)	
Malignant			
Carcinoma%	43.75% (7)	31.58% (12)	0.392
Sarcoma%	0.00% (0)	13.16% (5)	0.313

*Statistically significant (*P* < 0.05)

The preoperative data of the two groups, including demographics, smoking and drinking history, comorbidities, nature of tumor, except for sex, have no significant differences, which make the two groups comparable.

### Operation data

The FHL group had 16 patients. All the FHL were used to fill the dead space left by tumor resection. Fourteen patients of them used skin paddle to repair mucous defects. One case used FHL to repair mucous defect because of the unavailable skin paddle. One case used both FHL and skin paddle to repair mucous and skin defect. The nFHL group had 38 patients and used skin paddle to repair mucous defects.

The FHL group had 2 cases used for maxillary reconstruction and 14 cases for mandibular reconstruction. The nFHL group had 38 cases all for mandibular reconstruction. The rates of mandibular were 0.00 and 12.50%; there was no significant difference (*P* = 0.152). In neck dissection, the FHL group had 7 cases, 2 cases of which were bilateral neck dissection. The nFHL group had 13 cases, 2 cases of which were bilateral neck dissection. The neck dissection rates were 43.75 and 34.21%; there was no significant difference (*P* = 0.507). In donor site, the FHL group had 9 cases in the right, 7 cases in the left. The nFHL group had 8 cases in the right and 30 cases in the left. The rate of the left was 43.75 and 57.89%. The difference was significant (*P* = 0.011). The mean overall operation time was higher in the FHL group (11.90 ± 2.55 h vs 8.91 ± 1.29 h) than nFHL group. The difference was significant (*P* = 0.000). The mean flap harvesting time was 118.63 ± 11.76 min in the FHL group. It was shorter than nFHL group, 125.74 ± 11.33 min. The difference was significant (*P* = 0.042). The mean length of the fibula was 19.94 ± 2.67 cm in FHL group. The nFHL group was 19.8 ± 2.88 cm. There was no significant difference (*P* = 0.935). The median size of skin paddle is 16.5 (range 0–96) cm^2^ in FHL group. It was smaller than the nFHL group, 21(range 10–104) cm^2^. The difference was significant (*P* = 0.027). Two cases in FHL group received skin grafting and 3 cases in nFHL group (see Table 2).

**Table 2:** Operation data

	FHL group	nFHL group	*P*
Site			
Maxilla%	12.50% (2)	0.00% (0)	0.152
Mandible%	87.50% (14)	100.00% (38)	
Neck dissection%	43.75% (7)	34.21% (13)	0.507
Bilateral ND%	12.5% (2)	7.89% (3)	0.985
Donor side			
Left%	43.75% (7)	78.95% (30)	0.011*
Right%	56.25% (9)	21.05% (8)	
Skin-grafting%	12.5% (2)	7.89% (3)	0.985
Operation time (hour)	11.90 ± 2.55	8.91 ± 1.29	0.000*
Flap harvesting time (min)	118.63 ± 11.76	125.74 ± 11.33	0.042*
Fibula length (cm)	19.94 ± 2.67	19.87 ± 2.88	0.935
Skin paddle size (cm^2^)	16.5 (0–96)	21.0 (10–104)	0.027*

*Statistically significant (*P* < 0.05)

### Postoperative data

The median total hospital days of FHL group were 21.5 (range 18–37) days. The nFHL group was 25.5 (range 15–71) days. There was no significant difference (*P* = 0.095). The median postoperative hospital days of FHL group was 12 (range 8–27) days, nFHL group was 12.5 (range 6–27) days. There was no significant difference (*P* = 0.696). The mean hospitalization expenses of FHL group were 71.29 ± 18.37 thousand yuan; nFHL group was 72.50 ± 19.57 thousand yuan. There was no significant difference (*P* = 0.833). We observed the patients’ recovery condition after operation. All the flaps survived completely with the overall success rate of 100%. No vascular crisis happened in FHL group, and one case of vascular crisis happened in nFHL group. All patients were satisfied with the treatment and esthetics effect. In terms of perioperative complications, the main complication was infection. There were 5 cases of infection in the FHL group and 8 cases in the nFHL group. The infection rates were 31.25 and 21.05%. There was no significant difference (*P* = 0.651) (see Table 3).

**Table 3:** Postoperative data

	FHL group	nFHL group	*P*
Hospital days (day)	21.5(18–37)	25.5(15–71)	0.095
Postoperative hospital days (day)	12.0(8–27)	12.5(6–27)	0.696
Hospitalization expense (yuan)	71.29 ± 18.37	72.50 ± 19.57	0.833
Infection%	31.25%(5)	21.05%(8)	0.651

### Donor-site morbidity

As for donor-site morbidity, we had observed the morbidity of the FHL group. And as a contrast, we chose 16 patients of the nFHL group, of which the operation date was close to the FHL group. We thought that the follow-up time of the two groups was accordant. Eleven patients in FHL group and 12 patients in nFHL group received follow-up. Five patients in FHL group and 4 patients in nFHL group lost to follow-up because of being out of touch. The median follow-up time was 20 months (range 3–42 months) in FHL group. And the nFHL group is 16 months (range 3–41 months). Among 11 patients in FHL group, 9 patients were satisfied with the recovery of the feet. Among 12 patients in nFHL group, 10 patients were satisfied with the recovery of the feet. In the FHL group, 3 cases had gait abnormality, 3 cases had claw toe, 2 cases had chronic pain, 2 cases had edema, and 1 case had weakness. In nFHL the group, 7 cases had claw toe, 4 cases had sensory deficit, 1 case had chronic pain, 1 case had gait abnormality, and 1 case had edema.

Above all, comparing the two groups, there were significant differences in the operation time, flap harvesting time, and skin paddle size. There were no significant differences in hospital days, postoperative hospital days, hospitalization expense, and rate of perioperative complications.

Compared with FFF without FHL, FFF-FHL will neither affect the use of flap nor bring more time cost, economic burden, and perioperative complications. The donor-site morbidity was slight.

In the operation, some emergency situations may happen. Here are two cases using FHL-FFF to repair the maxillary and mandibular extensive defect when dealing with the emergency situations.

#### Case 1

Case 1 is a male, 65 years old, having squamous cell carcinoma of upper gingiva.

**Fig. 3:**
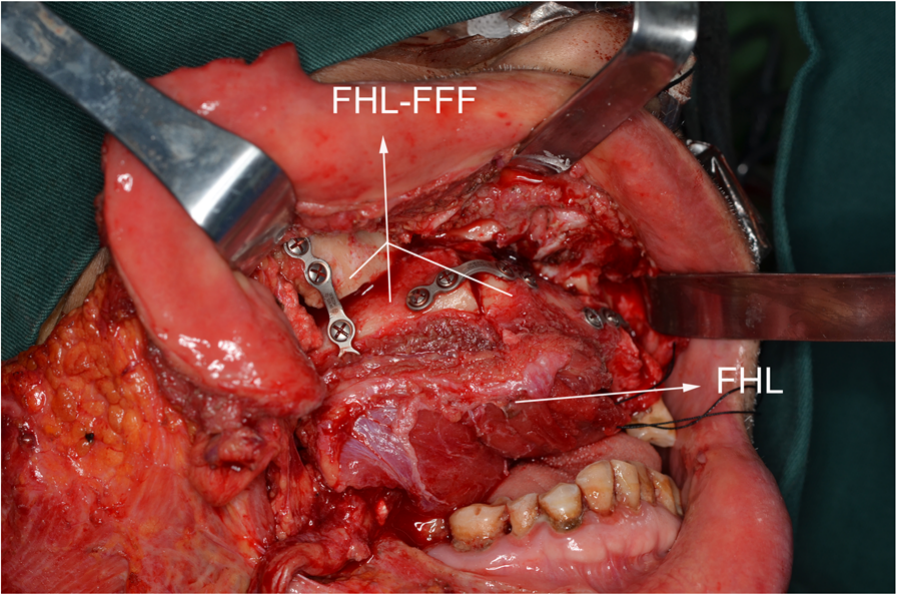
FHL-FFF in repairing maxillary extensive defect: the FHL for intraoral defect

**Fig. 4:**
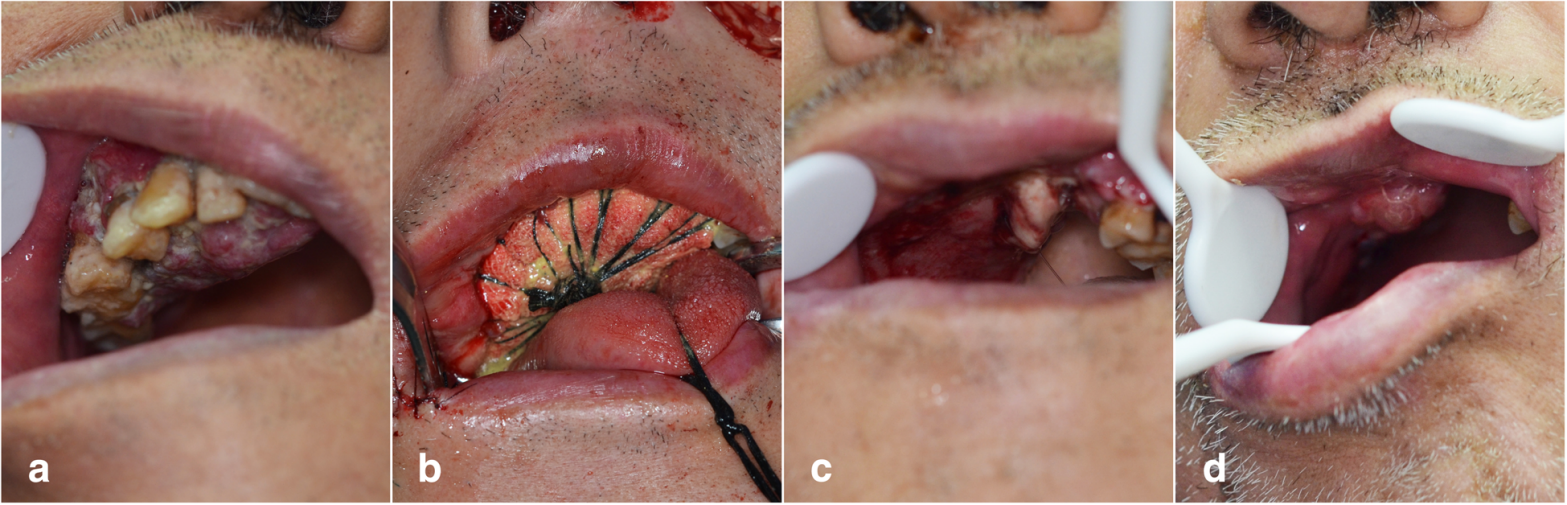
Case 1: intraoral mucosa forming. **a** Pre-operation. **b** During operation. **c** One week after operation: the FHL formed good oral mucosa. **d** One month after operation

The emergency situation in flap harvesting was the perforator vessel variation. When seeking perforator, we found that the perforator was not from the peroneal artery. It resulted in the unavailability of the skin paddle. The patient had both bone defect and soft tissue defect. We had to abandon the unavailable skin paddle and seek substitution to replace it. The operator flexibly used the FHL to close the intraoral defect (Fig. 3). The FFF was without skin paddle. To accelerate the intraoral mucosa forming, we used an artificial biological membrane to cover the myofascial surface of FHL and used an iodoform cotton wrapping for pressing. The skin paddle was sutured in situ. One week later, when we removed the iodoform cotton wrapping, the intraoral mucosa recovered well. (Fig. 4a–d) And the fibular flap survived and had no infection and necrosis. The patient was satisfied with the appearance and oral functional recovery.

#### Case 2

Case 2 is a male, 67 years old, having squamous cell carcinoma of lower gingiva.

**Fig. 5:**
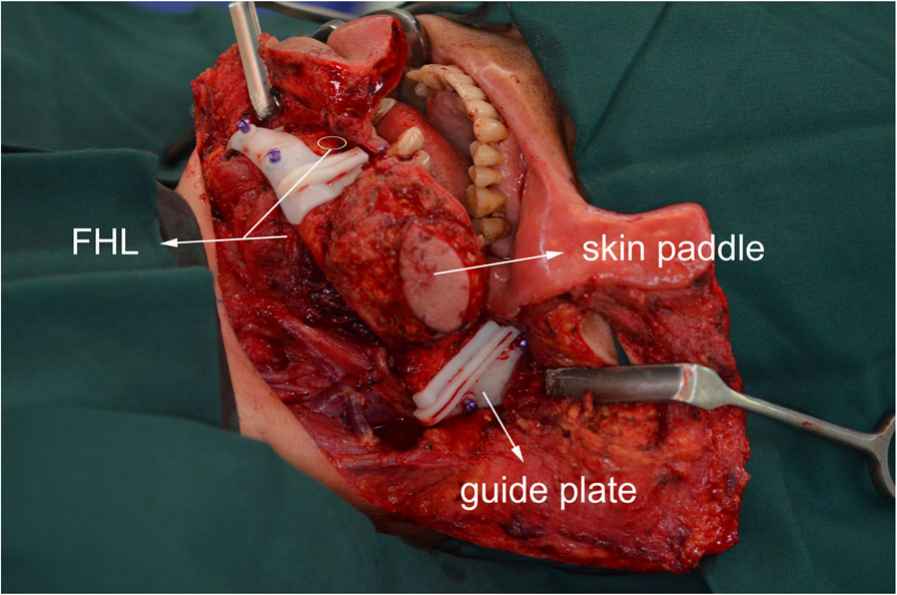
FHL-FFF in repairing mandibular extensive defect: the FHL for intraoral defect and skin paddle for extraoral defect

**Fig. 6:**
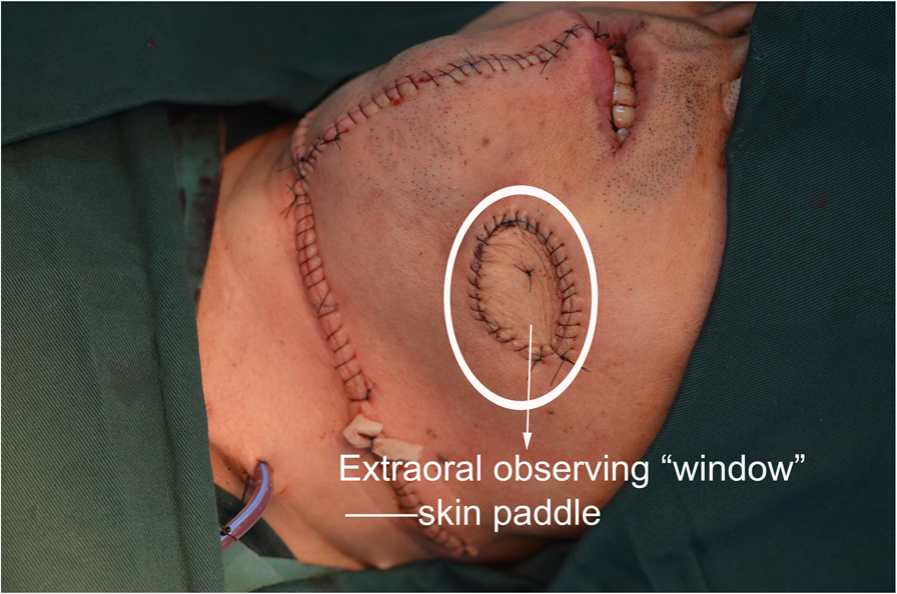
Extraoral observing “window”: skin paddle

**Fig. 7:**
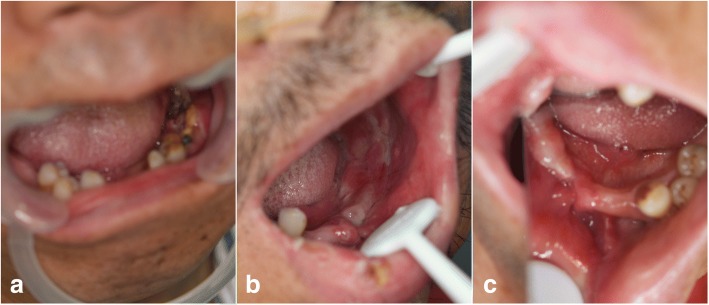
Case 2: intraoral mucosa forming. **a** Pre-operation. **b** Two weeks after operation: the oral mucosa recovered well. **c** Three weeks after operation

The emergency situation in flap harvesting was not enough flap tissue volume. Beyond our expectation, the tumor violated the facial skin. When we removed the violated facial skin, the skin paddle could not meet the intraoral defect and facial defect simultaneously. The operator flexibly used skin paddle repair facial defect and used it as a extraoral “window” that made it convenient to observe flap survival (Figs. 5 and 6). The FHL was used to repair the intraoral defect. And in case 1, we used an artificial biological membrane and an iodoform cotton wrapping. Ten days later, the intraoral mucosa recovered well (Fig. 7a–c). And the fibular flap survived and had no infection and necrosis. The patient was satisfied with the appearance and oral functional recovery.

## Discussion

The maxillary and mandibular extensive defects bring huge challenge in the reconstruction. Some epithelial benign tumors, such as ameloblastoma, will sometimes leave extensive osseous and soft tissue defects after tumor excision. The epithelial malignant tumors will accompany more extensive defects. The conventional FFF, consisting of fibula and skin paddle cannot fully fill the dead space after tumorectomy. In order to repair the extensive defects, we need to increase the size of skin paddle. When the size is more than 4 to 6 cm, it is difficult to suture the incision. It will increase the risks of wound dehiscence and compartment syndrome if we try to suture the incision [8, 9]. In 1986, Hidalgo mentioned that the FHL muscle can help fill the dead space and repair the defects [10].

The conventional FFF relies on skin paddle to repair soft tissue defect. The perforator vessel variation limits the use of skin paddle. Some skin paddles may be unavailable because of the miss of perforator vessel. Although FFF in the contralateral leg can be harvested, it will increase the damage and operation time. In our department, there was once a patient had to undergo another FFF because of the perforator of one leg was not found. In our study, we used a FHL-FFF without skin paddle to deal with perforator vessel variation.

The skin paddle has hair follicle and subcutaneous fat. Some patients feel annoyed because of the hair growing. The hair and wrinkled skin make it hard to stay clean in oral. The heavy subcutaneous fat has a poor influence on chewing and try-in of denture base and dental implant. The volume of skin paddle often needs to be reduced to make denture and implant try-in smoothly [9]. In dental implant, the establishment and maintenance of a soft tissue seal around the transmucosal part of an implant is vital for implant treatment’s success [11]. The skin paddle does not provide an appropriate peri-implant environment [11, 12]. It results in peri-implant mucositis and peri-implantitis and reduces lifetime of dental implant [11]. In our study, we used FHL to form good oral mucosa.

Summarized above conventional FFF’s deficiencies, we find that the not enough flap tissue volume, perforator vessel variation, and poor oral mucous epithelization are the top three problems. To resolve these problems, we add the FHL in FFF.

To cope with the not enough flap tissue volume, we can harvest a FHL-FFF in advance for the preparation. In case 2, we harvested the FHL-FFF before the tumor resection. Owing to the FHL-FFF, we could repair the intraoral and extraoral defect simultaneously. To resolve the not enough tissue volume, some researchers raised the double and triple skin paddle FFF to reconstruct defects [13–15]. The multi-skin paddle FFF may be a good solution for multi-defect of jaw. But it may increase operation time and the difficulty of operation. The harder close of donor site’s incision and the uncertain perforators also limit the use of multi-skin paddle FFF.

To cope with the perforator vessel variation, we can seek perforators firstly. If the perforators cannot be used, we can immediately change the operation plan and turn to harvest a FHL-FFF. In case 1, if we did not have any preparation, the flap harvesting may fail because of perforator vessel variation. It proved that FHL-fibula flap could be survived without a skin paddle. A FFF without skin paddle can no doubt avoid the shortcoming of a skin paddle, while it loses the function of observation window of the skin paddle.

The two cases have formed good mucosa. It suggests that the FHL-FFF can resolve the problem of poor oral mucous epithelization. The use of artificial biological membrane is to protect the FHL from being exposed and accelerate mucous epithelization. The good mucosa is also helpful for the dental implant. When using the FHL for good mucosa, the FHL covers on the fibula and muscular fasciae of FHL repair the mucosal defect. If we want to implant, we may need to reduce the muscle thickness. It needs further study cooperating with Oral Implantology Center.

The FFF with FHL has been reported since early time. In 1992, Schusterman et al. recommended that a cuff of soleus and flexor hallucis longus be incorporated into the flap to help ensure flap viability in using the osteocutaneous fibula flap [16]. In 1994, Hidalgo introduced that flexor hallucis longus muscle lay conveniently under the fibula to fill in the soft tissue defect in mandible reconstruction [4]. And in 1995, Hidalgo mentioned that flexor hallucis longus muscle was anatomically convenient for obliterating dead space in a review of 60 consecutive fibula free flap mandible reconstruction [10]. In 1997, Ruch et al. used the fibula-flexor hallucis longus osteomuscular flap to reconstruct a massive defect in limb salvage [17]. These were the early application of FHL-FFF. In 2001, Cho et al. studied the blood supply of osteocutaneous free fibula flap and found that it was recommended that a soleus and flexor hallucis longus muscle cuff be included to incorporate these perforators when designing an osteocutaneous free fibula flap 10 to 20 cm from the fibular head [18]. From Schusterman et al. and Cho et al.’s research, we can learn that the FHL incorporated into the flap can help raise the reliability of perforators. In 2002, Schoeller reported that they applied the reinnervated fibula-flexor hallucis longus free flap in the functional recovering and reconstruction after the Ewing sarcoma excision [19]. The function of upper limb recovered well. It proves that FHL-FFF has become a popular source of vascularized bone and skin for limb reconstructions. In 2003 and 2011, Peng and Mao et al. reported the use of free fibula-flexor hallucis longus myofascial flap in maxillary reconstruction [9, 20]. They found that the flexor hallucis longus myofascial flap could replace the skin paddle and improve the shortage of the skin paddle. In their research, they used the flexor hallucis longus myofascial flap to repair the intraoral defect and the mucosa totally formed after 3 month. In our study, FHL has been proved to be able to repair the intraoral defect and the use of artificial biological membrane can accelerate the myofascial flap’s mucosa forming. About 10 days after surgery, it can be found the mucosa totally forms. Through enough time to observe, the mucosa of FHL’s surface is steady and safe (see Figs. 3a–d and 6a–c).

To review our control study, the carcinoma rate of FHL group was higher than nFHL group. Although the difference was not significant, in some degree, it suggests that the FHL-FFF may be better for large carcinoma. The overall operation time depends on multi factors, such as neck dissection. Although it is higher significantly in FHL group, in the flap harvesting time, it is shorter significantly in FHL group. The proportion of neck dissection, especially bilateral neck dissection, in FHL group was higher, which also suggests the FHL-FFF is better for large carcinoma. It suggests that the FHL-FFF can reduce the flap harvesting time. The reason is that we do not need to dissect the peroneal artery and FHL. So, the harvesting procedure is simplified. The size of skin paddle in FHL group was smaller significantly. It suggests that the FHL-FFF can reduce the dependency of skin paddle and reduce the loss of the leg skin. There were no significant differences in total hospital days, postoperative hospital days, and hospitalization expenses. It suggests that the FHL-FFF does not increase time cost and economic burden compared with FFF (without FHL). There was no significant difference in the rate of perioperative complications. It suggests that the FHL-FFF does not lead to more perioperative complications. As for the long-term complications, such as donor-site morbidity, it needs more follow-up study. In our present study, the donor-site morbidity is slight. Most patients were satisfied with the recovery of the feet. The more reliable results about morbidity depend on more patients and longer follow-up time.

The advantages of FHL-FFF are as follows: FHL can increase the volume of soft tissue and help fill the dead space and repair the defects. The FHL-FFF can simplify the flap harvesting operation and save time. The FHL can make FFF rely less on skin paddle and replace the skin paddle in some cases. The FHL incorporated into the flap can help raise the reliability of perforators. The FHL-FFF can form good mucosa.

The indication of FHL-FFF is an extensive bone defect with moderate soft tissue defect if we only use the FHL to fill dead space. If we want to use the muscular fasciae of FHL to repair the intraoral defect and form oral mucosa, the indication is more strict: an extensive bone defect with moderate soft tissue defect as well as limited mucosa defect. The reason is that the movability of FHL is poor because of the blood supply. For this reason, the FHL-FFF is not suitable for folding. The folding will have a bad influence on the blood supply of FHL and increase the risk of necrosis.

The influence of removing FHL on donor-site function still needs further study. The FHL helps curve the ankle joint and hallux. The removing of FHL may have some influence on FHL function. Sassu said that nerve injury to the FHL muscle is unlikely during fibula flap harvest [21]. In 2014, van den Heuvel reported that free fibula flap donor site morbidity in terms of hallux function is independent of the inclusion or exclusion of the FHL muscle in the flap [22]. Another prospective study about the influence on patients’ quality of life with/without FHL in the flap has been performed by us. The data at present suggests that the FHL-FFF has not brought more functional loss and more complication. We believe more reliable conclusions will be reached soon.

Overall, the retrospective study weakens the strength of this study. The limits of this study are obvious. The population is too small. The follow-up study is barely satisfactory. The case of maxillary defect is too small. In our further study, we will add more patients and raise reliability of the study.

## Conclusions

FHL-FFF can be flexibly used in the reconstruction of the maxillary and mandibular extensive defect. FHL-FFF can provide more tissue volume and be used in larger carcinoma and emergency situation for perforator vessel variation as well as forming good mucosa. Furthermore, the simple and convenient flap harvesting makes it fitter for younger surgeons to conduct.
